# How to formulate high-quality lessons learned: a rapid review

**DOI:** 10.1080/16549716.2025.2546691

**Published:** 2025-11-12

**Authors:** Christian Dagenais, Michelle Proulx, Aurélie Hot, Esther McSween-Cadieux, Romane Villemin, Lara Gautier, Sydia Rosana de Araujo Oliveira, Patrick Cloos, Lola Traverson, Kate Zinszer, Valéry Ridde

**Affiliations:** a Department of Psychology, Université de Montréal, Montreal, QC, Canada; b Écoles des sciences de l’administration, Université TÉLUQ, Montréal, Canada; c Department of Psychology, Université du Québec à Montréal, Montreal, QC, Canada; d School of Public Health, Université de Montréal, Montreal, QC, Canada; e Fundação Oswaldo Cruz, Instituto Aggeu Magalhães, Recife, Brasil; f École de travail social, Université de Montréal, Montréal, QC, Canada; g Université Paris Cité, IRD, Inserm, Ceped, Paris, France

**Keywords:** Rapid review, quality lessons learned (QLLs), Quebec, hospitals’ resilience, COVID-19 pandemic project

## Abstract

Lessons learned convey information and experiences that were studied when carrying out projects or policies, in order to improve procedures and practices to better cope with future similar problems in other contexts. Although the term *lessons learned* appears in the titles of thousands of scientific articles, most do not describe how these lessons were produced or the level of rigor involved in their development. As part of a project aimed at deriving lessons from hospitals’ resilience during the COVID-19 pandemic in five countries (the HoSPiCOVID project), we sought to systematised the process of producing these lessons. To do so, we conducted a rapid review to identify the best ways of developing quality lessons learned (QLLs). A QLL results from a systematic process of collecting, compiling, and analysing data derived from a research project. The rapid review follows the same key steps as a systematic review, adapted to a more accelerated and pragmatic format. From 1,881 documents initially identified, 18 were retained. Their analysis identified three principles to guide the process of developing QLLs: 1) Creating a supportive climate; 2) Choosing the right leaders or facilitators for the process; and 3) Engaging in a scientific approach. Based on these findings, we developed a guide comprising 11 steps, structured into two main phases: preparatory steps for QLL development, and steps for identifying and formulating QLLs. This guide offers a structured process for teams seeking to enhance the rigor, clarity, and potential transferability of the lessons they formulate.

## Paper Context

- **Main findings:** This rapid review of quality lessons learned offers a proposed procedure for formulating QLLs.
- **Added knowledge:** The procedure is intended to be useful and applicable and warrants testing and evaluation.
- **Global health impact for policy and action:** It was aimed at supporting the resilience of hospital systems as part of the HoSPiCOVID project involving several research teams in different countries.

## Background

In early 2020, the Director of the World Health Organization (WHO) declared the emergence of a novel coronavirus a public health emergency of international concern [1]. As the outbreak rapidly escalated into the COVID-19 pandemic, a wide range of policies and practices were introduced to slow transmission, develop and validate rapid diagnostic tests, treat infected individuals, and create vaccines. Hospitals worldwide had to devise strategies to manage the influx of patients.

Amid the widespread disruptions caused by COVID-19, health systems around the world faced the urgent challenge of implementing effective control measures while maintaining essential services. In response, WHO emphasised the importance of systematically documenting lessons learned from national COVID-19 responses [2].

However, scientific studies rarely follow a structured process like the one described by WHO. Many articles present a set of lessons learned or feature the term in their titles, yet few explain the underlying processes through which these lessons were identified [3]. In practice, the scientific literature seldom details systematic, transparent, and rigorous approaches to identifying lessons learned [3–5].

This gap highlighted the need for clearer guidance on how to formulate quality lessons learned in a way that supports transferability and evidence-informed decision-making. In the context of research projects conducted in complex or rapidly evolving environments, there was a need for structured approaches that allow research teams to generate lessons grounded in empirical data and transparent reasoning.

This article presents the findings of a rapid review of best practices, procedures, and approaches for developing what Michael Q. Patton refers to as *quality lessons learned* (QLLs) [6] (see definition below).

The review was conducted as part of the HoSPiCOVID project, which compared the resilience of hospital and public health systems and their personnel in five countries (Brazil, Canada, Japan, France, and Mali) [7]. In addition to its empirical focus, the project also aimed to generate and share QLLs with hospital and public health officials in the participating countries in order to support future preparedness and system strengthening efforts.

## Method

A rapid review provides an overview of the available knowledge on a given object of study [8–10]. The method follows the same steps as a systematic review, but in an accelerated and simplified manner [11,12], to produce evidence within the shorter time frame imposed by an emergency situation [13,14], as was the case here. For this review, our team chose to limit the number of databases queried, excluding any assessment of the methodological quality of the selected papers and focusing on a descriptive synthesis of findings [14–16].

### Search strategy

The search strategy (see Appendix A) was developed in close collaboration with a senior information specialist. Search terms were related to: 1) lessons learned (excluding best practices) and 2) method (e.g. process, approach, criteria). The information specialist conducted a systematic search of the published and grey literature across two scientific databases: MEDLINE and Web of Science. MEDLINE was selected as it is the most widely used database in the health sciences. To complement the MEDLINE search and broaden the scope to related scientific fields relevant to the health sector, Web of Science was also used. We considered that the combination of these two databases provided adequate coverage of the health sciences literature for the purposes of this rapid review.

Grey literature was identified through searches using the Google Web search engine and consultations with experts. Google’s advanced search tool was employed to retrieve documents – particularly guides, tools, and similar resources – centred on the lessons learned process as the main topic, while minimising irrelevant results. This search yielded 215 relevant documents, which were added to the initial set.

### Study selection: eligibility criteria

Documents were included in the review if they: described a methodology or a process to capture quality lessons learned or presented a tool or a guide to capture lessons learned; were published between 2000 and 2020; and were published in English.

Documents were excluded from the review if they: discussed a process or a tool specific to a field that was not relevant to the health sector (e.g. computer science, engineering); discussed lessons learned from a single project or initiative (without describing how they produced them); or were not available in full text (e.g. abstract only).

### Data extraction: selection and coding

First, titles and abstracts of all documents were screened in Zotero by the first author to assess their potential eligibility based on the inclusion and exclusion criteria. Second, all documents presumed to meet the criteria were retrieved as full texts. The final selection of relevant documents was done independently by three authors (CD, MP, EMC) based on a full article review. Discrepancies concerning the retrieval of articles were resolved through consensus. Data were extracted by four authors (CD, MP, EMC, RV) using predefined Excel data extraction sheets, which included document characteristics as well as the criteria defined above.

Information extracted from the selected documents included:

- Characteristics of the selected items (e.g. title, authors, year of publication, country, journal, discipline);
- Definition proposed for ‘lesson learned’;
- Steps to produce lessons learned (e.g. timing, process, stakeholders);
- Methods or techniques for collecting data (e.g. interview grid);
- Quality criteria for a lesson learned (e.g. checklist);
- Methods of formulating lessons learned;
- Implementation or dissemination of lessons learned.

## Results

**Figure 1.**
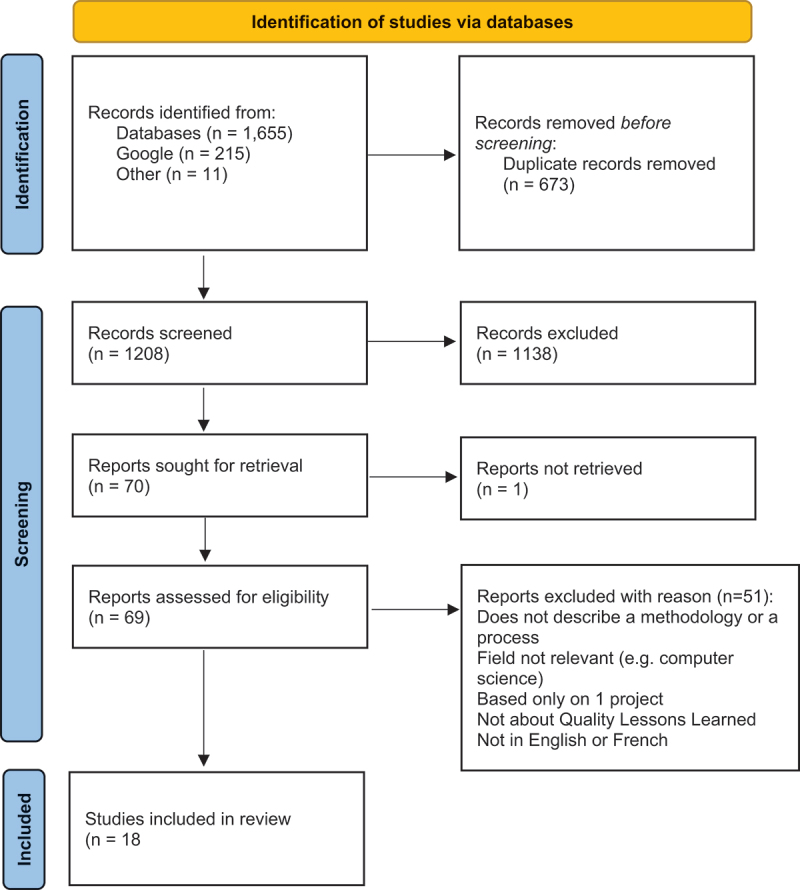
PRISMA flow-chart.

The search yielded 1,881 records, of which 1,208 were screened after removing duplicates. Eighteen documents published between 2000 and 2020 were included in the final review (Figure 1 - PRISMA flowchart). These documents were produced primarily in high-income countries: United States (5), United Kingdom (4), Canada (4), with others from Australia, Switzerland, Saudi Arabia, and collaborative efforts involving Japan and the UK or the US. They included peer-reviewed articles (6), grey literature from official bodies (5), monographs (2), a book chapter, and various other sources (e.g. commentaries, conference proceedings). A detailed list is available in Appendix B.

Despite their diversity, these documents converge on a common goal: improving how organisations generate and use lessons learned. They offer guiding principles, conceptual models, and procedures for producing what we refer to as ‘quality lessons learned’ (QLLs). Through a content analysis of this literature, we identified three core dimensions:

1. A shared definition of QLLs;
2. Guiding principles for their development;
3. A recommended set of steps to structure the process.*

### What is a Quality Lesson Learned (QLL)?

A *quality lesson learned* (QLL) is a statement or recommendation based on tacit or explicit knowledge, drawn from positive or negative experiences, and intended to guide future practice. QLLs aim to be transferable, helping others perform tasks more effectively or avoid repeating past mistakes.

Unlike anecdotal reflections, QLLs result from systematic efforts to collect, compile, and analyze information. They often emerge from evaluations of projects, programs, policies, or research activities. Their value lies in contextualising what happened, understanding why it happened, and distilling that insight into actionable guidance.

Several sources define QLLs as useful knowledge gained through experience—knowledge that can be generalised, shared, and applied in other settings [3,5,17–19]. They are most effective when they lead to concrete improvements in processes, efficiency, or safety [18,20].

### Guiding principles for the lessons learned development process

Three core principles emerged from the reviewed literature to guide the development of QLLs. Together, they highlight the importance of rigor, inclusion, and trust throughout the process.

#### Foster a safe and collaborative environment

Trust is essential for stakeholders to openly share both successes and failures [21]. Creating a climate of collaboration – across organisational levels – encourages honest reflection [5,22]. This requires clear communication, respect for all viewpoints, and transparency about the process and its benefits [23]. Support from leadership and shared responsibility among partners can further strengthen engagement [5,22].

#### Ensure credible and inclusive leadership

Effective facilitation depends on leaders who model openness, honesty, and respect [23]. Involving team or project leads from the start helps coordinate efforts, encourage participation, and ensure follow-up [18]. Managers can also play a key role in motivating teams and maintaining momentum.

#### Apply a systematic, evidence-informed approach

The QLL development process should follow a structured, transparent methodology grounded in a research mindset. This includes formulating a guiding question, collecting data systematically from multiple sources, and analysing it rigorously and collaboratively [24]. Primary and original data sources are preferable. Over-reliance on a single perspective or selective data filtering should be avoided to preserve the credibility of findings.

### Steps for developing quality lessons learned

Drawing on the 18 selected documents, we propose an **11-step iterative approach** for developing and disseminating quality lessons learned (QLLs). These steps are organised into two key phases:

#### Phase 1 – preparing the process (steps 1–5)

This phase lays the foundation for a credible, inclusive, and rigorous QLL process.

- **Step 1: Identify and engage stakeholders**
  Stakeholders – internal and external – should be involved early to clarify goals, scope, data sources, and organisational constraints [5,18,20,25]. Their involvement ensures that the QLLs are relevant and actionable.
- **Step 2: Define a clear objective**
  The process should have a clearly stated purpose, co-developed with stakeholders. Are the QLLs meant to inform internal reflection, external sharing, future planning, or all of these? [3,20,26]
- **Step 3: Specify the project(s) and events under review**
  Clarifying the project or event of interest (e.g. crisis response) allows teams to define the scope, responsibilities, timeline, and frequency of analysis [4,20,27].
- **Step 4: Choose the right timing**
  QLLs can be developed at various points – not just post-project. Capturing lessons during implementation may enhance ongoing effectiveness and reduce memory bias [5,17,20,22,27–29].
- **Step 5: Select a data collection strategy**
  The approach should be adapted to the audience, scope, time frame, available resources, and roles of those involved [18,20,26]. It should rely on diverse and credible data sources.

#### Phase 2 – identifying and formulating QLLs (steps 6–11)

This phase focuses on data gathering, analysis, validation, and dissemination.

- **Step 6: Define data collection questions**
  Common guiding questions include: *What happened? What worked? What didn’t? What should be done differently?* [5,20,21,26,29,30]. These may be explored through After Action Reviews (AARs) or other structured group discussions [18,23,30].
- **Step 7: Collect data**
  Data sources may include group discussions, individual interviews, documents, direct observation, and incident reports [18,21,23,24,26,28,30]. Triangulation helps ensure completeness.
- **Step 8: Verify data and fill gaps**
  Before analysis, teams should identify missing elements and collect any additional information needed to complete the picture [26].
- **Step 9: Analyse data and formulate QLLs**
  QLLs should be grounded in a systematic analysis – whether through team discussions, content analysis, analytical frameworks, or conceptual models (e.g. Syllk model, triple-loop learning) [3,4,17,21,24,27–29]. Analytical tools (e.g. matrices or grids) can support consistency (Table 1).

**Table 1.** Proposed grids for QLL analysis.

Authors	Analysis grid
CDC [17]	Project information and contact information for additional detail A clear statement of the lesson A background summary of how the lesson was learned Benefits of using the lesson and suggestions for how it may be used in the future
McDonald [24]	Analysis of the data collected by relevant category (environment, inputs, process, outcomes, etc.) using the following questions: what happened, what contributed to success, what contributed to failure, what needs to be improved? In-depth examination of the data for the ‘process’ category using a congruence assessment matrix (see Appendix C)
Milton [18]	Identify what went well and what could have gone better Identify the audience for whom the QLL is intended Revisit the target situation or context Analyse the differences between what was expected and what happened Look for the root causes, i.e. create a list of causes Make a recommendation for the future.
Qatar National Project Management (n.d.) [31]	Purpose Project goal Project outcomes Project evaluation (this section should state whether or not the project’s outcomes were realised and describe how success was measured against outcomes) Project strengths Project areas for improvement Advice for similar projects

- **Step 10: Validate QLLs**
- Validation ensures that QLLs are accurate, relevant, and useful. This may involve structured feedback from stakeholders or review sessions with senior actors [4,6,18,29]. Criteria include contextual clarity, specificity, supporting evidence, target audience relevance, and applicability conditions [4,6,24,32,33].

- **Step 11: Archive, disseminate, and implement QLLs**
- Lessons should be stored in accessible formats (databases, web portals), shared across networks (e.g. newsletters, communities of practice), and integrated into ongoing training and project planning [3,5,18,20,22,26,28,29,34]. Implementation requires leadership, follow-up, and sometimes a roadmap to ensure that lessons lead to concrete change.

## Discussion

### Purpose and scope of the guide

This article presents the foundation of a practical and evidence-informed guide for producing quality lessons learned (QLLs), structured around three guiding principles and an 11-step process. The approach is intended to support teams plan a reasoned and rigorous process, while offering resources for deeper exploration of specific steps. By synthesising best practices from a diverse body of literature, the framework aims to generate lessons that are not only contextually grounded but also transferable and actionable – ultimately improving organisational learning and future responses.

### Distinctive contribution

Unlike much of the existing literature, which provides fragmented or anecdotal recommendations [3,5], this guide proposes a comprehensive, step-by-step method grounded in three guiding principles: creating a climate of trust, selecting appropriate leadership, and adopting a scientific approach. This structure enhances both conceptual clarity and practical implementation.

### Empirical support and flexibility

As mentioned earlier, the guide was developed and applied in the context of the international HoSPiCOVID project, conducted across five countries with varying levels of resources. This application demonstrates the framework’s flexibility and relevance in both crisis situations and routine quality improvement initiatives [7]. While many authors emphasise high-risk contexts for lessons learned, others highlight the value of capturing insights from everyday operations [22,35]. This dual focus reinforces the guide’s relevance for both adaptive and everyday resilience.

### Filling a gap in literature and practice

The guide addresses a widely acknowledged gap: although thousands of articles reference ‘lessons learned,’ few describe how these are derived or whether they are transferable. In contrast, this framework emphasises data triangulation, stakeholder engagement, and contextual analysis [4], offering a transparent and replicable process for formulating QLLs. The sharing of tools, guiding questions, and examples in the rapid review ensures adaptability to diverse contexts and sectors.

### Addressing limitations and critical issues

Despite its contributions, the literature examined in this rapid review presents several shortcomings. Few publications describe in detail the analytical steps used to transform collected information into quality lessons learned (QLLs). As such, more effort is needed to systematise and specify the procedures that lead to QLLs – such as identifying problems, documenting resolutions, analysing challenges, and formulating evidence-based recommendations.

Moreover, the actual application and use of QLLs are rarely addressed in a systematic manner. Similarly, few authors critically reflect on the influence of power dynamics among stakeholders – yet these relationships inevitably shape how experiences are interpreted, potentially leading to resistance or facilitation of change.

Another important gap concerns the role of those initiating or leading the process. Their perspectives may unconsciously shape the way QLLs are framed – for instance, through confirmation bias [36] or cognitive bias [24]—by favouring information that aligns with their expectations and worldview. While the QLL approach proposed here incorporates mechanisms to mitigate these risks (e.g. source triangulation, seeking disconfirming evidence, and validation exercises), these biases remain a potential concern – especially when the evaluators are internal actors with institutional stakes in the outcomes.

Finally, interpersonal or organisational tensions must not be overlooked. As previously mentioned, acknowledging conflict and valuing collaboration among individuals with diverse perspectives is essential for producing well-rounded and robust lessons [36].

### Policy and system-level implications

Beyond individual projects, the guide has clear implications for public policy and health systems. The 11-step process has proven adaptable in low- and middle-income countries, as illustrated in the HoSPiCOVID project across a range of settings, providing initial support for its adaptability. We recommend that this structured, participatory approach be proactively integrated into policy cycles – from design to implementation – to strengthen learning, accountability, and adaptive capacity [6,18]. Ministries, public agencies, and international organisations could embed the protocol within evaluation and quality assurance mechanisms.

### Comparison with traditional quality improvement approaches

While traditional quality improvement (QI) methods – such as Plan-Do-Study-Act (PDSA) cycles – prioritise rapid, pragmatic solutions rooted in experiential knowledge, they often lack generalisability. In contrast, the QLL approach is grounded in research logic, combining tacit and explicit knowledge, rigorous data collection, stakeholder validation, and transparent synthesis. This makes it particularly valuable in research settings where generalisation, accountability, and knowledge mobilisation are essential.

Future research should further explore how the QLL approach can complement or integrate with existing QI practices, especially in resource-constrained environments. It is also necessary to establish clearer criteria for distinguishing a QLL from a personal opinion or anecdote. While valuable lessons may emerge from individual experiences, they must be supported by credible data, triangulated perspectives, and a transparent analytical process to be recognised as QLLs. Additional studies are needed to evaluate the implementation of the guide across various institutional and cultural contexts and to assess the quality, utility, and impact of QLLs on real-world decision-making.

## Conclusion

This rapid review proposes a structured, evidence-informed procedure for formulating quality lessons learned (QLLs), grounded in principles of transparency, stakeholder engagement, and methodological rigor. Developed to support a multi-country project on hospital system resilience, the approach offers a practical tool to help teams reflect critically on their experiences and generate actionable insights.

Beyond this specific context, the guide addresses a broader gap in both literature and practice: while thousands of scientific articles refer to ‘lessons learned,’ few describe how these were derived or whether they are transferable. This work responds to that gap by offering a replicable, step-by-step method applicable to health systems research, organisational learning, and post-crisis evaluations.

The proposed procedure has already been implemented in the hospital resilience project, with initial results presented in a separate article. A second article, currently under review with *Global Health Action*, provides a detailed account of the implementation process [37].

Ultimately, this work supports researchers, practitioners, and decision-makers seeking to move beyond anecdotal observations towards the development of validated, high-quality lessons to inform future projects, policies, and organisational responses. Ongoing work will explore how the approach is used in practice and how it may evolve based on user feedback.

## Data Availability

The data that support the findings of this study are available from the corresponding author, CD, upon reasonable request.

## Preprint

A previous version of this article has been published as a preprint in SSRN: https://dx.doi.org/10.2139/ssrn.4255090.

## APPENDIX A – Search Strategy

**Project**: Rapid review on the question “How to develop quality lessons learned?”?

### Key words

**Table:**

Concept 1	Concept 2
Lessons learned	Process
Lessons learnt	Approach*
Learned lessons	Method*
Learn lessons	Framework*
Learning lessons	Model*
	Validat*
	Document*
	Identif*
	Conceptualis*/Conceptualiz*
	Formalis*/Formaliz*
	Develop*
	Captur*
	Formulat*
	Design*
	Checklist*
	Generat*
	Criteria
	Implement*
	Collect*
	Disseminat*
	Archiv*
	Apply*
	Based
	Shar*
	Tool*
	Structure/structures
	System/systems
	Manage*
	Evaluat*

### Requests

#### Web of Science

(AB=(((lesson NEAR/1 learn*) OR (lessons NEAR/1 learn*)) NEAR/2 (apply* OR based OR shar* OR tool* OR structure OR structures OR system OR systems OR manage* OR evaluat* OR guide*))

OR

AB=(((lesson NEAR/1 learn*) OR (lessons NEAR/1 learn*)) NEAR/2 (implement* OR collect* OR disseminat* OR archiv*))

OR

AB=(((lesson NEAR/1 learn*) OR (lessons NEAR/1 learn*)) NEAR/2 (process OR approach* OR method* OR framework* OR model* OR validat* OR document* OR identif* OR select* OR conceptualiz* OR conceptualis* OR formaliz* OR formalis* OR develop* OR captur*))

OR

AB=(((lesson NEAR/1 learn*) OR (lessons NEAR/1 learn*)) NEAR/2 (formulat* OR design* OR checklist* OR generat* OR criteria*)))

AND

(TI=(lesson* NEAR/1 learn*) OR AK=(lesson* NEAR/1 learn*))

### Medline

(((lesson adj1 learn*) or (lessons adj1 learn*)) adj2 (process or ‘approach*’ or ‘method*’ or ‘framework*’ or ‘model*’ or ‘validat*’ or ‘document*’ or ‘identif*’ or ‘select*’ or ‘conceptualiz*’ or ‘conceptualis*’ or ‘formaliz*’ or ‘formalis*’ or ‘develop*’ or ‘captur*’)).ab.

OR

(((lesson adj1 learn*) or (lessons adj1 learn*)) adj2 (‘formulat*’ or ‘design*’ or ‘develop*’ or ‘checklist*’ or ‘generat*’ or ‘criteria*’)).ab.

OR

(((lesson adj1 learn*) or (lessons adj1 learn*)) adj2 (‘implement*’ or ‘collect*’ or ‘disseminat*’ or ‘archiv*’)).ab.

OR

(((lesson adj1 learn*) or (lessons adj1 learn*)) adj2 (‘apply*’ or based or ‘shar*’ or ‘tool*’ or structure or structures or system or systems or ‘manage*’ or ‘evaluat*’)).ab.

OR

(((lesson adj1 learn*) or (lessons adj1 learn*)) adj2 guide*).ab.)

AND

(lesson* adj1 learn*).kw,ti.

***Google*** (the terms retained for the study are those that produced the most results in the databases)

allintitle:‘lessons learned’ process OR processes OR approach OR approaches OR model OR models OR framework OR frameworks OR guide OR guidelines OR management OR capturing OR identifying OR documenting OR conceptualising OR conceptualizing OR applying OR sharing OR implementing filetype:pdf after:2000–01–01

allintitle:‘lessons learnt’ process OR processes OR approach OR approaches OR model OR models OR framework OR frameworks OR guide OR guidelines OR management OR capturing OR identifying OR documenting OR conceptualising OR conceptualizing OR applying OR sharing OR implementing filetype:pdf after:2000–01–01

## APPENDIX B – Description of documents included in the rapid review and topics covered

**Table:**

Author(s)	Country	Type of documents	Topics covered
Bray et al., 2013	United Kingdom	Guide produced by the National Institute for Health Research	After action review (AAR) procedures
CDC (2006)	USA	Document produced by the Centers for Disease Control and Prevention (CDC)	Best practice guide – QLL Questions to ask Archiving
Cronin & Andrews (2009)	United Kingdom	Scientific article (procedures)	AAR procedure
Davies (2009)	United Kingdom	Working paper	Definition of a QLL Group discussion procedure Example of a QLL sentence Quality criteria Example of a QLL
Duffield & Whitty (2016)	Australia	Scientific article (action research)	Application of the Systemic Lessons Learned Knowledge (Syllk) model to organizational management Details on the application of the model developed by the authors
Friesen et al. (2017)	Canada	Scientific article (literature review)	Definition of a framework for sharing best practices when faced with emergencies or events, and for prioritizing recommendations
Lo & Fong (2011)	United Kingdom & Japan	Publication in conference proceedings (literature review, theory)	Proposed method for asking good questions to develop good knowledge and QLLs
Mansourian & Vallauri (2020)	Switzerland	Scientific article (literature review, theory)	Description of a model to develop QLLs in the context of forest restoration
McClory et al. (2017)	United Kingdom	Scientific article (theory, literature review)	QLLs drawn from project management Proposed new model for capturing QLLs during the lifetime of a project
McDonald (2015)	USA	Monograph	Method for identifying QLLs
McIntyre and Kaminska, 2011	Canada	Document produced by Defence Research and Development Canada – Centre for Security Science	After event review (AER) procedures Based on the OODA Loop model (Boyd, 1987). Six steps for developing QLLs
Milton (2010)	United Kingdom & USA	Monograph	Handbook on Lessons learned
Nova Scotia (n.d.)	Canada	Document produced by Nova Scotia Department of Health and Health Promotion and Protection	Grid to be completed to produce a QLL
Patton (2001)	USA	Scientific article	On evaluation, knowledge management, best practices for QLLs
Qatar National Project Management (n.d.)	Saudi Arabia	Document produced by Qatar National Project Management (QNPM)	Guide for preparing to present a QLL
Rowe & Sikes (2006)	USA	Scientific article (theory)	Presentation of different steps for developing QLLs
White & Cohan (2005)	USA	Document produced by the Nature Conservancy	Guide to support QLL development Dissemination strategy
Williams (2007)	United Kingdom	Document produced by the Vivace Consortium	Guide presenting a series of guidelines associated with generating and applying feedback processes

## APPENDIX C. – Table to guide the analysis (proposed by McDonald, 2015) [Question Matrix for Assessing Congruence within the Process Component of the System. [38,39]

**Table:**

DOMAIN	FORMAL ORGANIZATION	INFORMAL ORGANIZATION	NETWORK OF INDIVIDUALS	INFORMATION NETWORK	TASK
FormalOrganization		To what degree are the structures of the formal organization consistent with the behaviors in the informal organization?	To what degree does the formal organization make use of individual resources and meet individuals needs?	To what degree can the capacity of the formal organization’s communication system handle the flow and storage of information?	To what degree do the structures of the formal organization motivate task-relevant behavior and facilitate task completion?
Informal Organization	To what degree is the culture of the informal organization consistent with the goals and rewards of the formal organization?		To what degree does the informal organization make use of individual resources and meet individual needs?	To what degree are the relationships of the informal organization reflected in the information network?	To what degree do the relationships of the informal organization motivate task-relevant behavior and facilitate task completion?
Network of Individuals	To what degree do individuals support the formal organization’s goals and make use of the formal organization’s resources to support their own personal goals?	To what degree do individuals support the informal organization’s culture and make use of the informal organization’s relationships to support their own personal goals?		To what degree can the cognitive capacity of individuals accommodate the flow of information?	To what degree do individual skills and abilities match task demands?
Information Network	To what degree is the information network consistent with the structure of the formal organization?	To what degree does the information network accommodate the communication expectations of the informal organization?	To what degree does the information network provide individuals with the information they require?		To what degree does the information network communicate information relevant to the task?
Task	To what degree are the demands of the task compatible with and converge with th emission and functions of the formal organization?	To what degree are the demands of the task compatible with and converge with the relationships of the informal orgnanization?	To what degree does the task meet individual needs?	To what degree are the task’s requirements reflected in the flow and storage of information?	
